# *Aesculus hippocastanum* Extract Exerts Neuroprotective Effects in an MPP^+^‐Induced Parkinson's Disease Model via PPARγ Activation

**DOI:** 10.1111/jcmm.71006

**Published:** 2026-01-05

**Authors:** Sarah Adriana Scuderi, Alessio Ardizzone, Giovanna Casili, Deborah Mannino, Antonio Catalfamo, Marika Lanza, Emanuela Esposito

**Affiliations:** ^1^ Genetics and Pharmacogenetics Unit ‘Gaetano Martino’ University Hospital Messina Italy; ^2^ UniCamillus‐Saint Camillus International University of Health Sciences Rome Italy; ^3^ Department of Chemical, Biological, Pharmaceutical and Environmental Sciences University of Messina Messina Italy; ^4^ Research Operative Unit of Neuropharmacology and Translational Neurosciences, Oasi Research Institute, IRCCS Troina Italy

**Keywords:** *Aesculus hippocastanum*, neuroinflammation, oxidative stress, Parkinson's disease (PD), peroxisome proliferator activated receptor gamma (PPARγ)

## Abstract

Parkinson's disease (PD) is a progressive neurodegenerative disorder characterised by the loss of dopaminergic neurons in the substantia nigra. In this study, we investigated the neuroprotective and anti‐inflammatory potential of *Aesculus hippocastanum* (horse chestnut extract, HCE) in an in vitro model of PD. Human neuroblastoma SH‐SY5Y cells were treated with the neurotoxin 1‐methyl‐4‐phenylpyridinium (MPP^+^) (1 mM) and/or in combination with HCE at the concentrations of 15.6, 31.2 and 62.5 μg/mL for 24 h. After 24 h, several analyses have been performed. Treatment with HCE at the concentrations of 31.2 and 62.5 μg/mL significantly improved cell viability following MPP^+^‐induced neurotoxicity. Furthermore, HCE effectively modulated key Parkinsonian markers by restoring tyrosine hydroxylase (TH) and reducing the number of α‐syn‐positive cells. At the same concentrations, HCE also attenuated NF‐κB signalling pathway activation and diminished the release of pro‐inflammatory cytokines IL‐1β, IL‐17, and TNF‐α. Notably, HCE promoted the activation of the nuclear receptor peroxisome proliferator activated receptor gamma (PPARγ), known for its neuroprotective properties, and reduced both oxidative and nitrosative stress. Crucially, silencing of PPARγ abolished the beneficial effects of HCE, indicating that its neuroprotective actions are mediated specifically through PPARγ activation. Thus, these findings suggest that HCE confers neuroprotection in vitro by regulating inflammation and oxidative stress primarily via PPARγ modulation.

## 1 Introduction

Parkinson's disease (PD) is a common neurodegenerative disease worldwide impacting more than 10 million people [1]. It is characterised by the selective loss of dopaminergic neurons in the substantia nigra, leading to a significant decline in striatal dopamine levels [1, 2]. Clinically, PD manifests through a combination of motor symptoms such as bradykinesia and rigidity, and non‐motor symptoms, including depression, sleep disturbances, and cognitive decline [1]. A pathological hallmark of PD is the accumulation of intracellular inclusions known as Lewy bodies, primarily composed of aggregated α‐synuclein (α‐syn), a presynaptic protein involved in synaptic vesicle regulation [3]. In pathological conditions, α‐syn undergoes misfolding and aggregation, leading to the formation of toxic oligomers that trigger neuroinflammatory responses and neuronal death [4]. The accumulation of α‐syn aggregates has been shown to impair in turn tyrosine hydroxylase (TH) expression, the rate‐limiting enzyme in dopamine synthesis, disrupting dopamine biosynthesis [2].

While the exact aetiology of PD remains multifactorial, a growing body of evidence highlights the role of neuroinflammation as a critical driver of disease progression [2]. Microglia, the primary immune cells of the central nervous system (CNS), become activated in response to neuronal injury and pathological protein accumulation, such as α‐syn aggregates [5]. Upon activation, microglia release a host of pro‐inflammatory cytokines, chemokines, and reactive oxygen species (ROS), thereby perpetuating a cycle of neurotoxicity [6]. Central to this inflammatory cascade is the nuclear factor kappa B (NF‐κB) pathway, a transcription factor that regulates the expression of numerous genes involved in inflammation, immune responses, and apoptosis [7]. In the context of PD, sustained activation of NF‐κB has been implicated in exacerbating microglial responses and promoting neurodegeneration [5].

Conversely, in the last years, it has been demonstrated that the nuclear peroxisome proliferator‐activated receptor gamma (PPARγ) acts as an anti‐inflammatory transcription factor capable of attenuating NF‐κB signalling and promoting cellular homeostasis [8]. Activation of PPARγ has been shown to downregulate the expression of pro‐inflammatory cytokines and enhance neuronal survival [9, 10, 11]. Thus, therapeutic strategies aimed at suppressing NF‐κB and enhancing PPARγ activity represent a promising avenue for modulating neuroinflammation.

Recently, natural compounds with anti‐inflammatory and antioxidant properties have gained interest as potential neuroprotective agents [10, 12]. Among them, *Aesculus hippocastanum* (horse chestnut extract, HCE) has emerged as a promising candidate due to its rich phytochemical profile and the presence of escin as its main bioactive component which exhibits important anti‐inflammatory effects [13, 14]. Selvakumar et al. [15], demonstrated that escin provides neuroprotection in a mouse model of chronic MPTP/probenecid‐induced toxicity by regulating oxidative stress and apoptotic pathways, especially via modulation of Bcl‐2, Bax, Cyto‐C, and cleaved caspases. Similarly, Sun and colleagues [16] demonstrated that escin alleviated SH‐SY5Y neuronal injury induced by *Porphyromonas gingivalis* ‐derived lipopolysaccharide (Pg‐LPS) through its anti‐apoptotic effects.

Despite the well‐established therapeutic applications of HCE in vascular disorders and systemic inflammatory conditions [17, 18], its potential neuroprotective role in neuroinflammatory diseases, including PD, remains not fully investigated. Based on these considerations, the aim of the present study is to evaluate the therapeutic potential of HCE in reducing neuroinflammation and neurodegeneration in an in vitro model of PD. Specifically, we focus on elucidating how HCE modulates the NF‐κB and PPARγ signalling pathways, which play central roles in the regulation of inflammatory responses and neuronal survival. By characterising these mechanisms, we seek to determine whether HCE may represent a viable multi‐target natural compound for the development of novel neuroprotective strategies in PD.

## 2 Materials and Methods

### 2.1 Materials

HCE ( *Aesculus hippocastanum* L.) water‐soluble extract was kindly supplied by the Chemical Department of the University of Messina, and it was provided in the form of a dry powder.

HCE extract was prepared using a similar methodology used in previous studies [19, 20].

The qualitative and quantitative analysis of HCE was conducted using an HPLC‐DAD‐MS system, following established protocols present in the literature [19, 20] to ensure accurate profiling of saponin constituents. Chromatographic separation was performed using an Agilent Series HPLC system, coupled with a diode array detector and an ion trap mass spectrometer. Mass spectra were acquired in both positive and negative ion modes, within a mass‐to‐charge ratio range of 1065–1175 Da. Separation was achieved on a Poroshell 120 EC‐C18 column (4.6 × 100 mm, 2.7‐Micron; Agilent Technologies, Santa Clara, CA, USA), using a binary solvent system composed of methanol (solvent A) and water (solvent B), both containing 1 mM HCOONH_4_ and 1% HCOOH. The elution gradient was programmed as follows: 0 min, 70% A; 7 min, 100% A; held until 13 min; then returned to initial conditions by 13.1 min and held until 20 min. The flow rate was set at 0.3 mL/min, with a column temperature of 30°C. Identification of escin isomers was carried out through MSⁿ fragmentation patterns and confirmed by direct comparison with a certified β‐escin reference standard (U.S. Pharmacopeial Convention, 99% purity).

Quantification was based on a calibration curve constructed from six different concentrations of the reference compound, consistent with methodology reported in other studies [19].

HCE was prepared as a fine dry powder and stored under strictly controlled conditions, low humidity, constant temperature, and protection from light, in hermetically sealed containers with desiccants to prevent degradation.

The detailed profile of HCE, including the content of escin (19.55%), is provided in Table S1. Unless otherwise specified, all reagents and materials used in the experimental procedures were obtained from Sigma‐Aldrich.

### 2.2 Cell Line and Culture Conditions

The human SH‐SY5Y neuroblastoma cell line (ATCC CRL‐2266) was obtained from the American Type Culture Collection (ATCC) (Manassas, VA, USA). This cell line has the ability to differentiate into neuron‐like cells exhibiting morphological and biochemical characteristics of mature neurons upon treatment with retinoic acid (RA) (Sigma‐Aldrich, Milan, Italy). Cells were grown in Dulbecco's Modified Eagle Medium (DMEM) and Ham's F12 medium (1:1) (Sigma‐Aldrich; St. Louis, MO, USA), supplemented with 2 mM L‐glutamine (GlutaMAX, ThermoFisher Scientific Cat. No. 35050061; Waltham, MA, USA), 1 mM sodium pyruvate (Sigma‐Aldrich Cat. No. S8636; St. Louis, MO, USA), 10% fetal bovine serum (FBS) (Sigma‐Aldrich; St. Louis, MO, USA), and 1% penicillin/streptomycin (Sigma‐Aldrich Cat. No. P4333; St. Louis, MO, USA). Cells were maintained at 37°C in a humidified incubator with 5% CO_2_.

### 2.3 Cell Treatments

SH‐SY5Y cells (2 × 10^4^ cells/well) were seeded into 96‐well plates (Corning Cell Culture, Corning, NY, USA) and then differentiated with RA (100 nM) for 24 h. After 24 h, cells were treated with HCE at increasing concentrations (15.6, 31.2, 62.5, 125 and 250 μg/mL) for 24 h to identify higher concentrations with minimal cytotoxicity. The concentrations of HCE were chosen based on a previous study [21]. A stock solution of HCE (1 mg/mL) was prepared in basal medium.

After selecting the appropriate concentrations of HCE, SH‐SY5Y cells, following differentiation with RA (100 nM) for 24 h, were exposed to neurotoxin 1‐Methyl‐4‐phenylpyridinium (MPP^+^) (Sigma‐Aldrich) (1 mM) for an additional 24 h, either with or without HCE treatment, to simulate an in vitro model of PD [22].

### 2.4 Cell Viability Assay

After treatment, cell viability was evaluated through a mitochondria‐dependent colorimetric assay as earlier defined [23].

### 2.5 Immunoblotting

Western blot was performed in SH‐SY5Y cells as described before [23]. The following primary antibodies were used: NF‐κB (1:500; Santa Cruz Biotechnology, Dallas, TX, USA; sc‐8008 HRP), IκBα (1:500; Santa Cruz Biotechnology, Dallas, TX, USA; sc‐1643), and PPARγ (1:500; Santa Cruz Biotechnology, Dallas, TX, USA; sc‐271392). Glyceraldehyde‐3‐Phosphate Dehydrogenase (GAPDH) (1:1000; Santa Cruz Biotechnology; Dallas, TX, USA, sc‐365062 HRP) was used as the loading control. Image J was used to perform densitometric analysis.

### 2.6 TUNEL Assay

TUNEL (terminal deoxynucleotidyl transferase) In Situ Cell Death Detection Kit, TMR red (Roche Cat No. 12156792910) was performed. Images were captured at 40× magnification using a fluorescence microscope (Nikon Eclipse Ci‐L, NIKON CORPORATION, Tokyo, Japan).

### 2.7 Immunofluorescence

Immunofluorescence staining was performed as described before [24, 25]. The following primary antibodies were used: anti‐Tyrosine Hydroxylase (TH) (1:100, Cat. No. PA5‐85167, Invitrogen) and α‐syn (1:100, sc‐12767; Santa Cruz Biotechnology, Dallas, TX, USA). Images were captured at 40× magnification using a fluorescence microscope (Nikon Eclipse Ci‐L, NIKON CORPORATION, Tokyo, Japan).

### 2.8 Reactive Oxygen Species Quantification

Reactive Oxygen Species (ROS) Fluorometric Assay Kit (Assay Genie; Cat. No. MAES0112) was used according to the manufacturer's instructions. After seeding SH‐SY5Y cells in 12 well‐plates (3 × 10^4^ cells/well), cells were differentiated with RA (100 nM) for 24 h and then treated with MPP^+^ (1 mM), either with or without HCE and incubated at 37°C and 5% CO_2_. After 24 h of treatment, 2,7‐dichlorofuorescin diacetate (DCFH‐DA) (15 μM) was added to the cells and incubated for 1 h at 37°C. Cells were collected and resuspended in PBS for fluorescence detection (Ex/Em = 500 nm/525 nm) using the fluorescence microplate reader GloMax Discover (Promega).

### 2.9 Small Interfering RNA Conditions

SH‐SY5Y cells (1 × 10^5^ cells/well) were seeded on a 6‐well plate. After differentiation with RA for 24 h, cells were transfected with 50 nM small interfering RNA (siRNA) against PPARγ (#AM16708, siRNA ID 143093, Invitrogen, Waltham, MA USA) or 50 nM negative control (scrambled) siRNA (#AM4611, Invitrogen, Waltham, MA USA) for 6 h using Lipofectamine transfection reagent (#18324‐020, Invitrogen, Waltham, MA USA) according to manufacturers' instructions. After 6 h of incubation, SH‐SY5Y cells were treated with MPP^+^ (1 mM), alone or in combination with HCE (62.5 μg/mL) for an additional 24 h. After 24 h of treatment, western blot analysis and ELISA kits were performed.

### 2.10 ELISA Kits

The levels of 3‐Nitrotyrosine (Human 3‐NT ELISA Kit Elabscience; Cat. No. E‐EL‐0040), Cu/ZnSOD (Human ELISA Kit Invitrogen; Cat. No. BMS222), ROMO‐1 (Human ROMO‐1 ELISA kit, Aviva Systems Biology, Cat. No. OKEH01371), Glutathione (GSH) (GSH ELISA kit, Cloud‐Clone Corp, Cat. No. CEA294Ge), TNF‐α (Human TNF‐α Conferma ELISA Kit, Sigma Aldrich, Cat. No. EZHTNFA), IL‐1β (Human IL‐1β ELISA kit, Sigma Aldrich, Cat. No. RAB0273) and IL‐17 (Human IL‐17 ELISA kit, Cloud‐Clone Corp, Cat. No. SEA063Hu) were measured in SH‐SY5Y cell supernatant according to the manufacturer's instructions. TH and α‐syn were evaluated in SH‐SY5Y cell lysates (Human Tyrosine Hydroxylase ELISA kit, Antibodies‐Online.com, Cat. No. ABIN6960326; Human α‐Synuclein ELISA Kit, Invitrogen, Cat. No. KHB0061). The absorbance was measured at 450 nm.

### 2.11 NOx Assay

Nitrite assay was performed using Griess reagent as explained before explained [26].

### 2.12 Statistical Analysis

Following the evaluation of data distribution, statistical analysis was conducted using a one‐way ANOVA, with Bonferroni's post hoc test applied for multiple comparisons. Results are expressed as mean ± standard deviation (SD) from three independent experiments. GraphPad Prism version 9.00 was used. A *p*‐value of less than 0.05 was considered statistically significant.

## 3 Results

### 3.1 HCE Restores SH‐SY5Y Cell Viability Following MPP ^+^‐Induced Neurodegeneration

**FIGURE 1:**
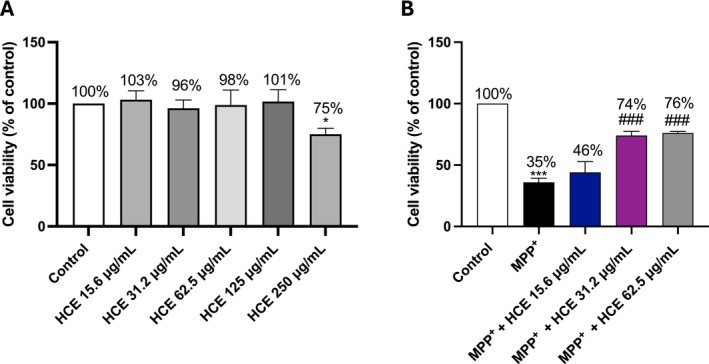
Evaluation of HCE cytotoxicity and neuroprotective effects. MTT assay was performed following treatment with HCE (15.6, 31.2, 62.5, 125, and 250 μg/mL) for 24 h (A) or co‐treatment with MPP^+^ (1 mM) and HCE (15.6, 31.2, and 62.5 μg/mL) for 24 h (B). Data are presented as mean ± SD from three independent experiments. One‐way ANOVA followed by Bonferroni post hoc test. *p < 0.05 versus Control, ***p < 0.001 versus Control; ^###^ *p* < 0.001 versus MPP^+^.

To identify non‐cytotoxic concentrations of HCE, SH‐SY5Y cells were treated with increasing concentrations of HCE (15.6, 31.2, 62.5, 125, and 250 μg/mL) for 24 h, and cell viability was assessed using the MTT assay (Figure 1A). No significant reduction in cell viability was observed up to 125 μg/mL (Figure 1A). A significant decrease in cell viability was detected only at 250 μg/mL (*p* < 0.05), indicating mild cytotoxicity at this highest concentration (Figure 1A), therefore it was excluded from subsequent analysis. Although the 125 μg/mL concentration was not cytotoxic, it was not included in further experiments as we chose to focus on lower concentrations that were already effective and clearly non‐toxic. This approach aimed to maximise biological relevance while minimising the risk of concentration‐dependent confounding effects.

Moreover, to investigate the neuroprotective effects of HCE, SH‐SY5Y cells were co‐treated with MPP^+^ (1 mM) and increasing concentrations of HCE (15.6, 31.2, and 62.5 μg/mL) for 24 h (Figure 1B). MPP^+^ alone induced a marked reduction in cell viability compared to the control group (Figure 1B; *p* < 0.001). Treatment with HCE at 31.2 and 62.5 μg/mL significantly improved cell viability compared to MPP^+^ alone (Figure 1B; *p* < 0.001), with the 62.5 μg/mL concentration showing the highest cell viability. The lowest concentration (15.6 μg/mL) produced a modest increase in cell viability that did not reach the minimal statistical significance (Figure 1B).

### 3.2 HCE Modulates PD Markers Levels Following MPP ^+^‐Induced Neurodegeneration

To evaluate the effect of HCE on PD‐related markers, we assessed TH and α‐syn in SH‐SY5Y cells by immunofluorescence.

**FIGURE 2:**
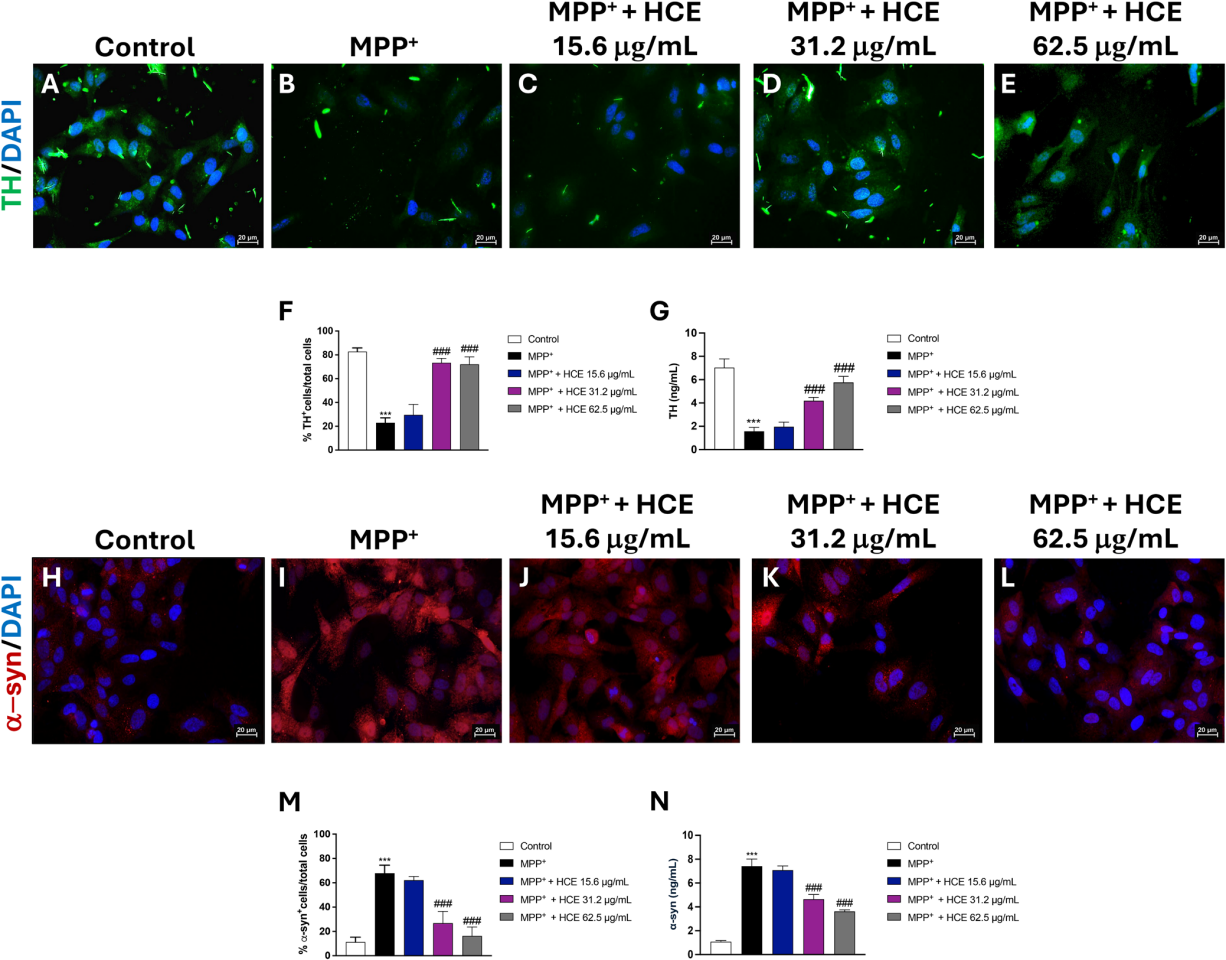
Effect of HCE on TH and α‐syn. Cells were treated with MPP^+^ (1 mM) and HCE (15.6, 31.2, or 62.5 μg/mL) for 24 h, then TH (A–F) and α‐syn positive cells (H–M) were evaluated by immunofluorescence. ELISA kits for TH and α‐syn were performed (G, N). Data are presented as mean ± SD from three independent experiments. One‐way ANOVA followed by Bonferroni post hoc test. ***p < 0.001 versus Control, ^###^ *p* < 0.001 versus MPP^+^.

Exposure to MPP^+^ (1 mM) significantly reduced the number of TH^+^ cells (Figure 2B, score 2F) compared to the control group (Figure 2A, score 2F).

Treatments with HCE attenuated these effects; indeed, HCE at 31.2 and 62.5 μg/mL significantly restored the number of TH^+^ cells compared to MPP^+^ alone (Figure 2D,E respectively, score 2F). The 15.6 μg/mL concentration had a slight but non‐significant effect on TH (Figure 2C, score 2F). The data about TH was confirmed by ELISA kit (Figure 2G).

Additionally, MPP^+^ markedly increased the number of α‐syn^+^ cells (Figure 2I, score 2M) compared to the control group (Figure 2H, score 2M), consistent with pathological protein accumulation typical of PD. Differently, both HCE at 31.2 and 62.5 μg/mL significantly reduced the number of α‐syn^+^ cells compared to MPP^+^ (Figure 2K,L respectively, score 2M), whereas the 15.6 μg/mL concentration produced only a modest, non‐significant decrease (Figure 2J, score 2M). This result was confirmed by the ELISA kit (Figure 2N).

### 3.3 HCE Modulates NF‐κB, IκBα and PPARγ Expression Following MPP ^+^‐Induced Neurodegeneration

To further investigate the mechanisms underlying HCE's neuroprotective effects, we assessed the expression of NF‐κB, IκBα, and PPARγ in SH‐SY5Y cells exposed to MPP^+^ and treated with increasing concentrations of HCE (15.6, 31.2, and 62.5 μg/mL) for 24 h.

**FIGURE 3:**
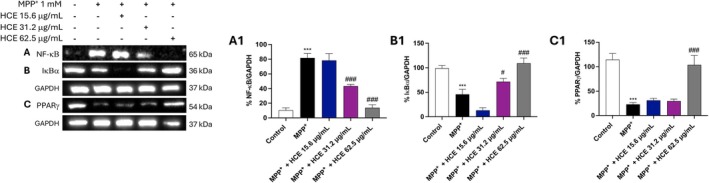
Effect of HCE on NF‐κB, IκBα, and PPARγ expression. Cells were treated with MPP^+^ (1 mM) and HCE (15.6, 31.2, or 62.5 μg/mL) for 24 h. (A, A1) MPP^+^ increased NF‐κB nuclear translocation, which was reduced by HCE at 31.2 and 62.5 μg/mL. (B, B1) IκBα expression decreased with MPP^+^ and was restored by HCE at 31.2 and 62.5 μg/mL. (C, C1) PPARγ expression was downregulated by MPP^+^ and significantly increased only by HCE at 62.5 μg/mL. Data are presented as mean ± SD from three independent experiments. One‐way ANOVA followed by Bonferroni post hoc test. ***p < 0.001 versus Control, ^#^ *p* < 0.05 versus MPP^+^, ^###^ *p* < 0.001 versus MPP^+^.

Treatment with MPP^+^ significantly increased nuclear translocation of NF‐κB compared to control group (Figure 3A, densitometric analysis 3A1), indicating activation of pro‐inflammatory signalling. Concurrently, IκBα expression was markedly reduced (Figure 3B, densitometric analysis 3B1).

Treatment with HCE inhibited NF‐κB activation and restored IκBα expression compared to MPP^+^ alone both at 31.2 and 62.5 μg/mL (Figure 3A,B respectively, densitometric analysis 3A1 and 3B1). The 15.6 μg/mL concentration showed a non‐significant effect compared to MPP^+^ (Figure 3A,B respectively, densitometric analysis 3A1 and 3B1).

Furthermore, MPP^+^ treatment led to a significant downregulation of PPARγ expression (Figure 3C; densitometric analysis 3C1). Among the concentrations tested, only HCE at 62.5 μg/mL significantly increased PPARγ expression compared to MPP^+^, while 15.6 and 31.2 μg/mL did not produce significant effects (Figure 3C; densitometric analysis 3C1).

### 3.4 HCE Reduces Pro‐Inflammatory Cytokines Levels Following MPP ^+^‐Induced Neurodegeneration

To assess the anti‐inflammatory potential of HCE, we measured pro‐inflammatory cytokines IL‐1β, IL‐17, and TNF‐α levels in SH‐SY5Y cell supernatants by ELISA kit.

**FIGURE 4:**
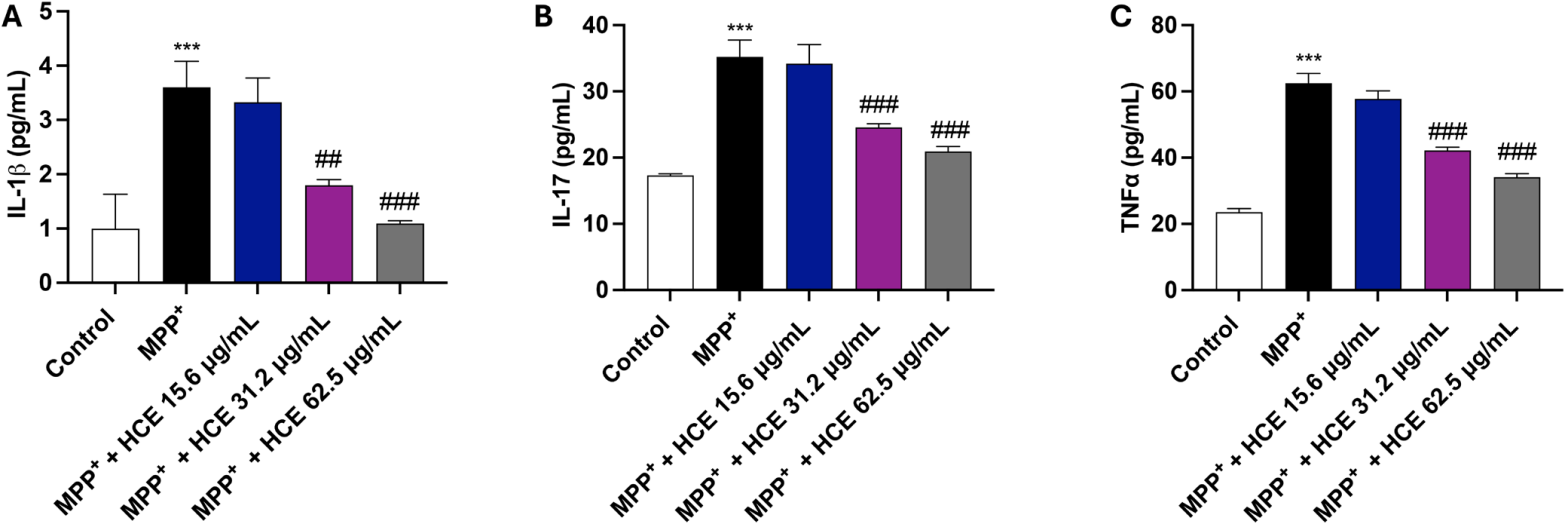
Evaluation of IL‐1β, TNF‐α, and IL‐17 levels. Cells were treated with MPP^+^ (1 mM) and HCE (15.6, 31.2, or 62.5 μg/mL) for 24 h. Cytokine levels were measured by ELISA kits in cell supernatants (A–C). Data are presented as mean ± SD from three independent experiments. One‐way ANOVA followed by Bonferroni post hoc test. ***p < 0.001 versus Control, ^##^ *p* < 0.01 versus MPP^+^, ^###^ *p* < 0.001 versus MPP^+^.

MPP^+^ treatment significantly increased the levels of all three cytokines compared to the control group (Figure 4A–C), indicating a robust inflammatory response following neurotoxic insult.

Co‐treatment with HCE attenuated the MPP^+^‐induced cytokine release in a concentration‐dependent manner. In fact, for all cytokines, significant reductions were observed only at both 31.2 and 62.5 μg/mL, with the highest concentration producing the strongest effect (Figure 4A–C). The 15.6 μg/mL concentration induced a slight non‐significant reduction (Figure 4A–C).

### 3.5 HCE Reduces Oxidative and Nitrosative Stress Following MPP ^+^‐Induced Neurodegeneration

**FIGURE 5:**
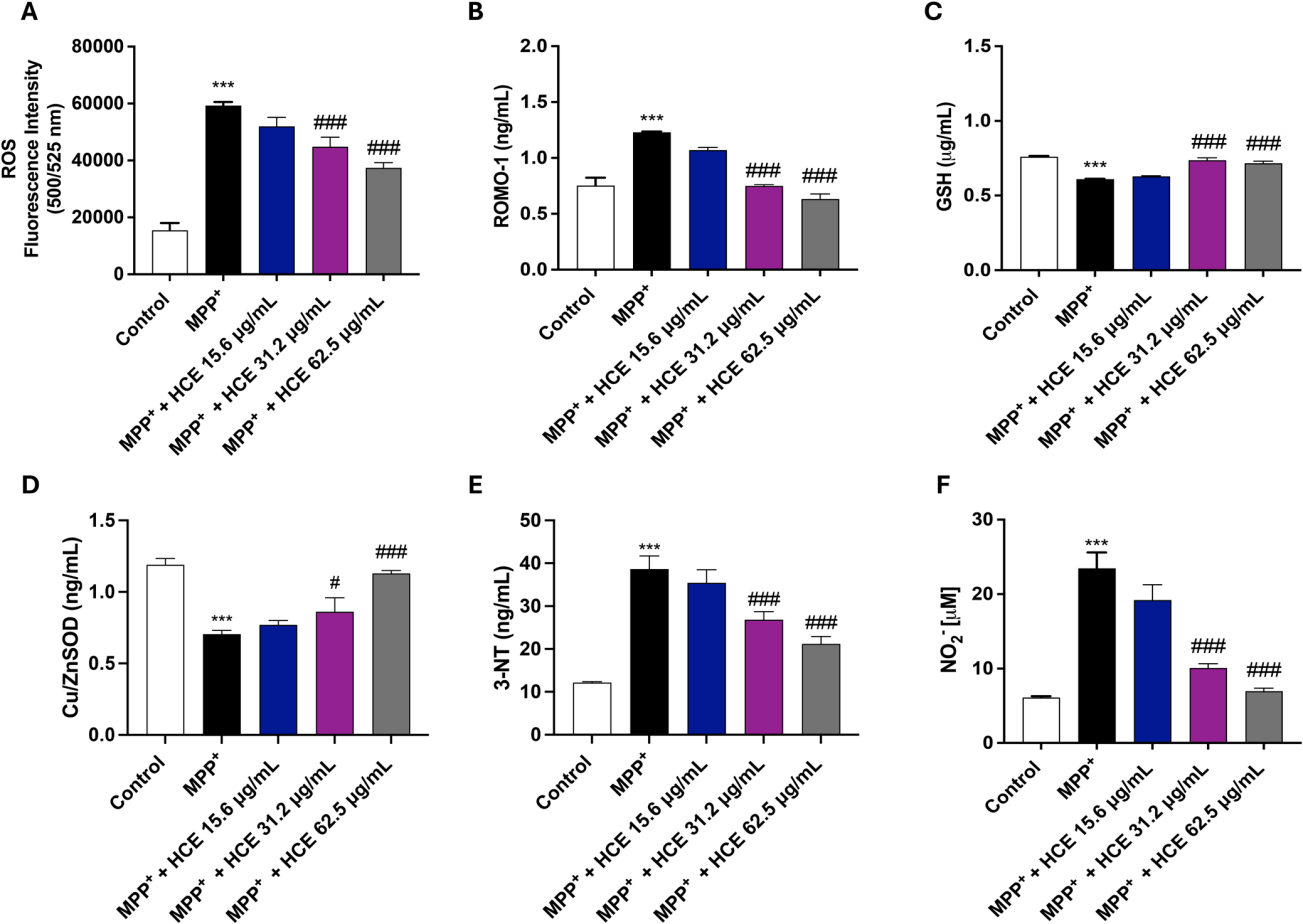
Evaluation of oxidative and nitrosative stress markers. Cells were treated with MPP^+^ (1 mM) and HCE (15.6, 31.2, or 62.5 μg/mL) for 24 h. Oxidative stress markers: (A) ROS, (B) ROMO‐1, (C) GSH, (D) Cu/ZnSOD. Nitrosative stress markers: (E) 3‐NT, (F) ${\mathrm{NO}}_{2}^{-}$. Data are presented as mean ± SD from three independent experiments. One‐way ANOVA followed by Bonferroni post hoc test. ***p < 0.001 versus Control, ^#^ *p* < 0.05 versus MPP^+^, ^###^ *p* < 0.001 versus MPP^+^.

To evaluate the antioxidant properties of HCE, we analysed markers of oxidative and nitrosative stress. In SH‐SY5Y cells, MPP^+^ increased the levels of ROS compared to the control group; however, treatment with HCE at the concentrations of 31.2 and 62.5 μg/mL demonstrated a reduction in them (Figure 5A). In addition, in SH‐SY5Y cell supernatants, MPP^+^ treatment significantly increased the levels of ROMO‐1 and reduced the levels of the antioxidant markers GSH and Cu/ZnSOD compared to the control group (Figure 5B–D), indicating a clear redox imbalance. Treatment with HCE at 31.2 and 62.5 μg/mL significantly reduced ROMO‐1 levels and restored GSH and Cu/ZnSOD levels (Figure 5B–D). The concentration of 15.6 μg/mL had minimal and non‐significant impact on all oxidative markers analysed (Figure 5A–D).

MPP^+^ exposure also led to increased levels of 3‐NT and ${\mathrm{NO}}_{2}^{-}$, displaying high nitrosative stress activation (Figure 5E,F). HCE at 31.2 μg/mL and 62.5 μg/mL significantly reduced both 3‐NT and ${\mathrm{NO}}_{2}^{-}$ levels, while no significant changes were observed at the concentration of 15.6 μg/mL (Figure 5E,F).

### 3.6 HCE Reduces Apoptosis Following MPP ^+^‐Induced Neurodegeneration

To assess whether HCE limits apoptotic cell death induced by MPP^+^, SH‐SY5Y cells were exposed to MPP^+^ (1 mM) alone or co‐treated with HCE (15.6, 31.2, and 62.5 μg/mL) for 24 h, and apoptosis was evaluated by TUNEL staining.

**FIGURE 6:**
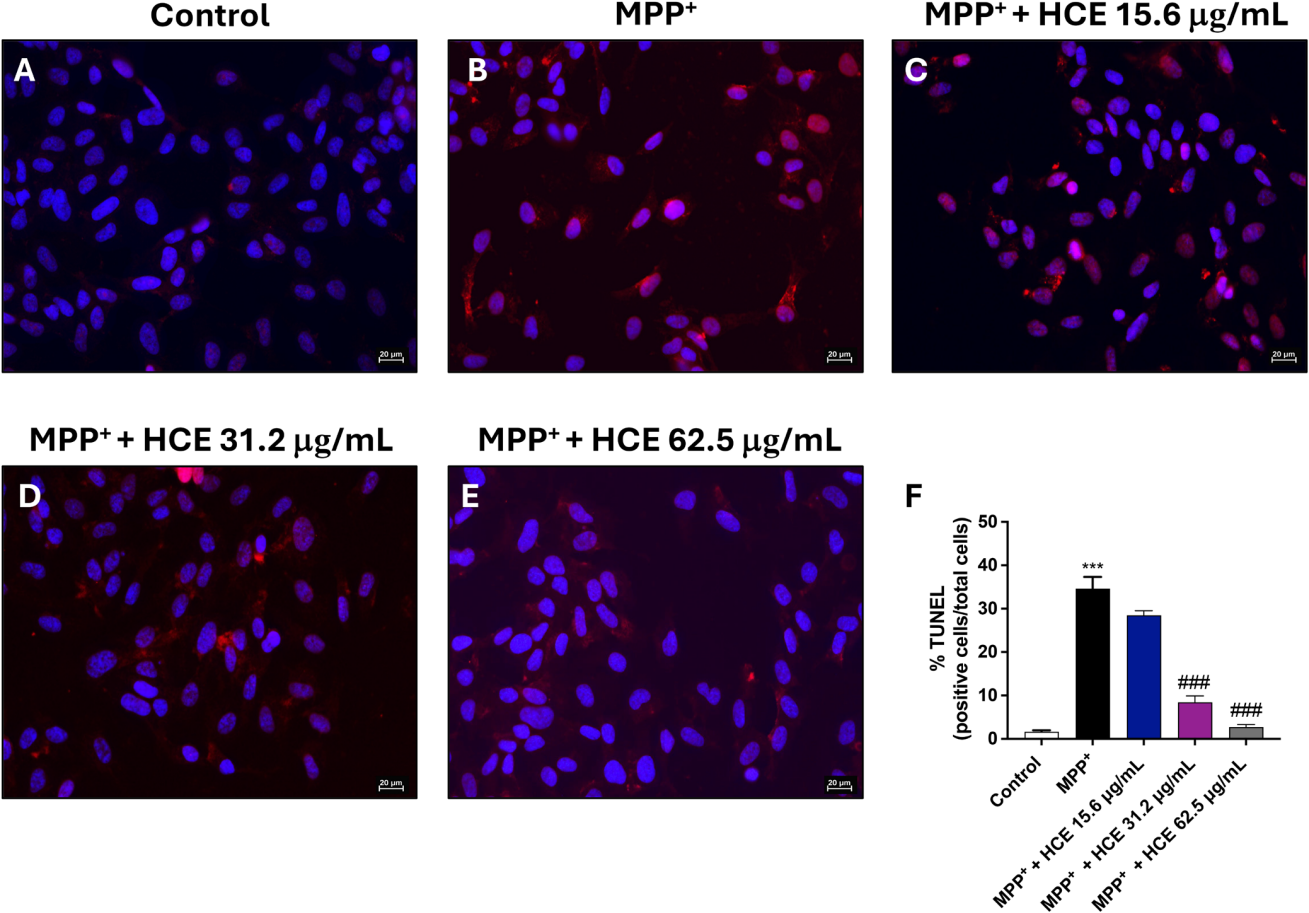
Assessment of apoptosis by TUNEL assay. Cells were treated with MPP^+^ (1 mM) and HCE (15.6, 31.2, or 62.5 μg/mL) for 24 h. (A–E) Representative TUNEL images. (F) Quantification of TUNEL‐positive cells was expressed as percentage. Data are mean ± SD of three independent experiments. One‐way ANOVA followed by Bonferroni post hoc test. ***p < 0.001 versus Control, ^###^ *p* < 0.001 versus MPP^+^.

Exposure to MPP^+^ significantly increased the percentage of TUNEL^+^ cells (Figure 6A, score 6F) compared to the control group (Figure 6B, score 6F), indicating extensive apoptosis. Co‐treatment with HCE at 31.2 and 62.5 μg/mL significantly reduced the percentage of TUNEL^+^ cells compared to MPP^+^ alone (Figure 6D,E, score 6F). The lowest concentration (15.6 μg/mL) yielded a modest, non‐significant decrease in apoptosis (Figure 6C, score 6F).

### 3.7 HCE Exerts Anti‐Inflammatory and Anti‐Nitrosative Effects Through PPARγ Activation

To fully investigate whether the anti‐inflammatory effects of HCE are mediated via PPARγ activation, SH‐SY5Y cells were transfected with PPARγ‐specific siRNA (siPPARγ) using Lipofectamine and subsequently treated with MPP^+^ (1 mM) and HCE (62.5 μg/mL) for 24 h. Scrambled siRNA was used as a negative control (siRNA NC).

**FIGURE 7:**
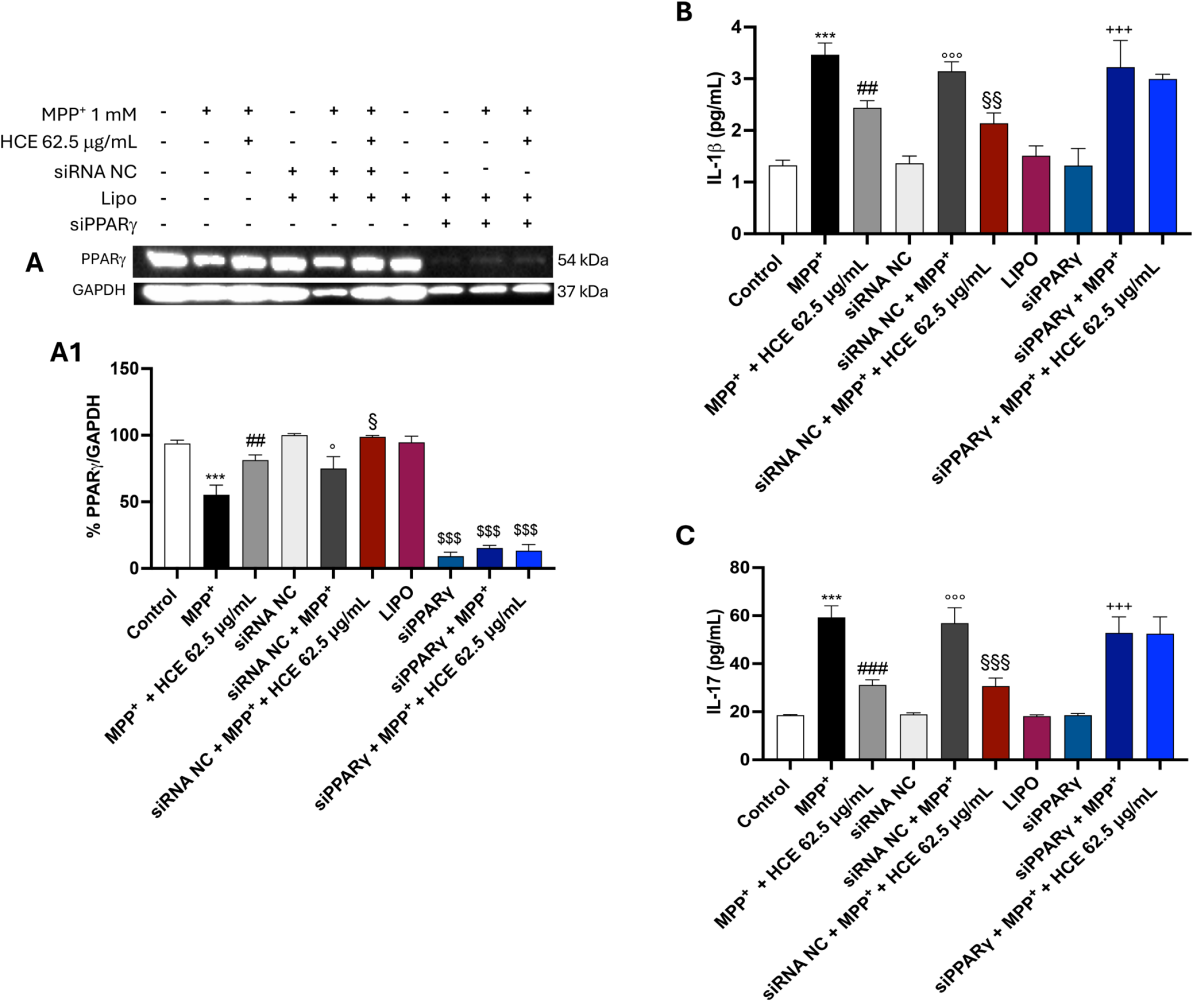
Evaluation of PPARγ involvement in HCE‐mediated anti‐inflammatory effects. SH‐SY5Y cells were transfected with scrambled siRNA (negative control; siRNA NC) or PPARγ‐targeting siRNA (siPPARγ) using Lipofectamine and treated with MPP^+^ (1 mM) and HCE (62.5 μg/mL) for 24 h. Protein expression of PPARγ (A; A1), levels of IL‐1β (B), and IL‐17 (C) were assessed. Data are presented as mean ± SD from three independent experiments. One‐way ANOVA followed by Bonferroni post hoc test. ***p < 0.001 versus Control, ^##^ *p* < 0.01 versus MPP^+^. ^###^ *p* < 0.001 versus MPP^+^, °*p* < 0.05 versus siRNA NC, °°°*p* < 0.001 versus siRNA NC, ^§^ *p* < 0.05 siRNA NC + MPP^+^, ^§§^ *p* < 0.01 siRNA NC + MPP^+^, ^§§§^ *p* < 0.001 siRNA NC + MPP^+^, ^$$$^ *p* < 0.001 versus Control, ^+++^ *p* < 0.001 siPPARγ.

In non‐silenced cells, HCE significantly increased PPARγ expression (Figure 7A, densitometric analysis 7A1) and reduced the levels of the pro‐inflammatory cytokines IL‐1β and IL‐17 (Figure 7B,C) compared to MPP^+^ alone. In contrast, in PPARγ‐silenced cells, the effects of HCE were completely abolished: PPARγ expression remained suppressed (Figure 7A, see densitometric analysis 7A1), and both IL‐1β and IL‐17 levels (Figure 7B,C) were not reduced by HCE treatment, remaining comparable to MPP^+^‐only conditions.

Moreover, to assess also if the antinitrosative effect of HCE depends on PPARγ activation, we measured 3‐NT and ${\mathrm{NO}}_{2}^{-}$ levels following cell silencing.

**FIGURE 8:**
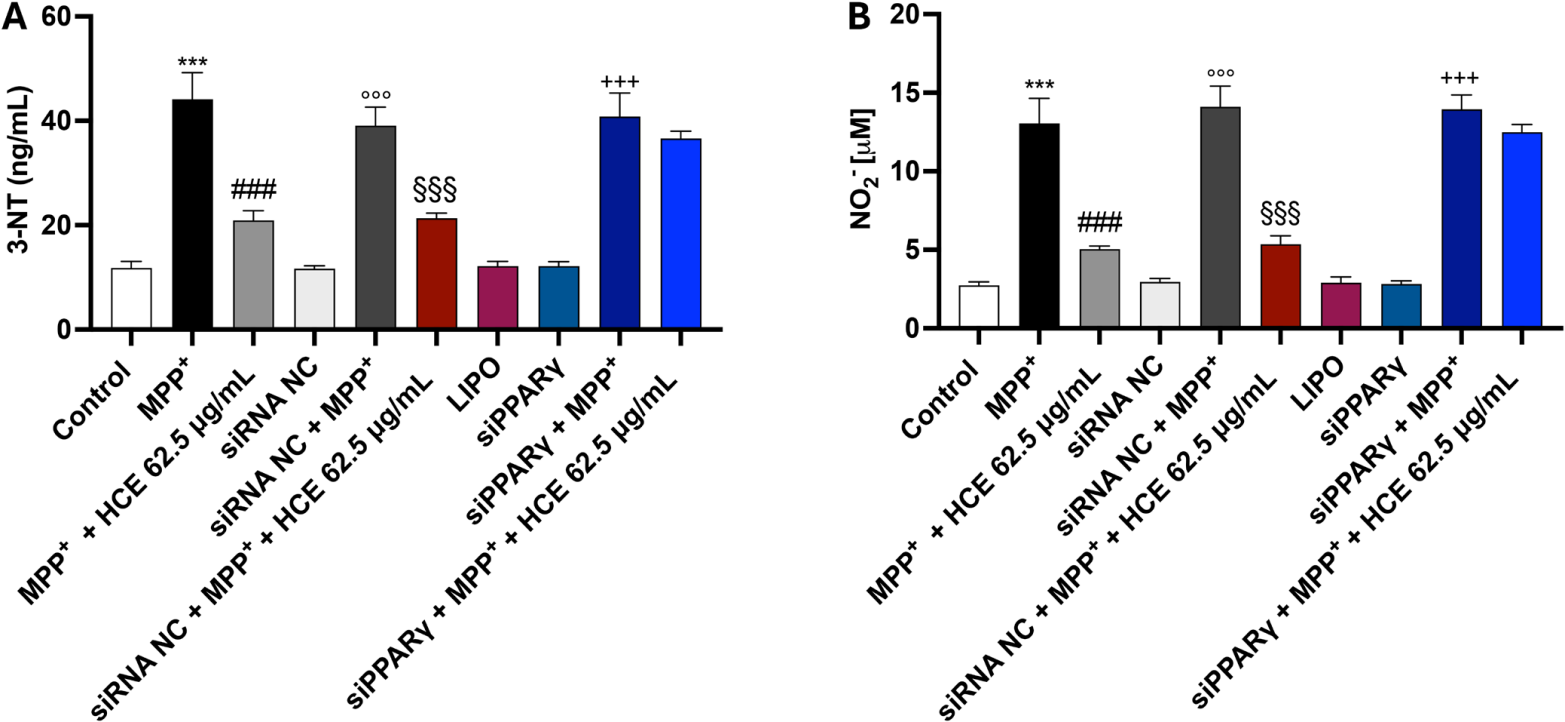
Evaluation of nitrosative stress markers following PPARγ silencing. Cells were transfected with scrambled siRNA (negative control; siRNA NC) or PPARγ siRNA using Lipofectamine and treated with MPP^+^ (1 mM) and HCE (62.5 μg/mL) for 24 h. Levels of (A) 3‐NT and (B) ${\mathrm{NO}}_{2}^{-}$ were measured in cell supernatants. Data are presented as mean ± SD from three independent experiments. One‐way ANOVA followed by Bonferroni post hoc test. ***p < 0.001 versus Control; ^###^ *p* < 0.001 versus MPP^+^; °°°*p* < 0.001 versus siRNA NC; ^§§§^ *p* < 0.001 siRNA NC + MPP^+^; ^+++^ *p* < 0.001 siPPARγ.

In non‐silenced cells, HCE significantly reduced both 3‐NT and ${\mathrm{NO}}_{2}^{-}$ levels compared to MPP^+^ alone (Figure 8A,B), confirming its antinitrosative action. However, in siPPARγ‐transfected cells, HCE failed to reduce either marker: 3‐NT and ${\mathrm{NO}}_{2}^{-}$ levels remained elevated and comparable to those in MPP^+^‐only conditions (Figure 8A,B).

## 4 Discussion

PD is a progressive neurodegenerative disorder [2]. Existing therapies often lose effectiveness over time, leaving patients increasingly affected by both motor and non‐motor symptoms. This therapeutic gap highlights an urgent need for novel disease‐modifying strategies that go beyond dopamine replacement and target the underlying pathophysiological mechanisms of the disease [2].

In the last decade, there has been a notable surge in research focusing on natural compounds for the management of PD [13, 27].

In this context, the present study aimed to examine the neuroprotective potential of HCE in an in vitro model of PD induced by MPP^+^. Given the well‐known antioxidant and anti‐inflammatory properties of this extract [14, 15], the present study further explored the underlying mechanisms, with a particular focus on the activation of PPARγ, a nuclear receptor increasingly recognised for its role in neuroinflammation and neurodegeneration [8, 11]. By elucidating this pathway, the study sought to assess whether HCE could represent a viable candidate for future therapeutic development in PD.

Chronic neuroinflammation is widely acknowledged as a contributing factor in the development and progression of PD [28]. Inflammatory responses within the CNS can compromise neuronal survival and exacerbate neurodegenerative processes, especially in vulnerable regions such as the substantia nigra [29]. This pro‐inflammatory environment is believed to interact with oxidative stress and other pathological mechanisms, ultimately amplifying neuronal damage [30]. As such, attenuating neuroinflammation has become a relevant focus in the search for neuroprotective strategies.

In this study, MTT assay provided a first indication of the cytoprotective effects exerted by HCE in the PD cellular model. As expected, exposure to MPP^+^ significantly reduced the viability of SH‐SY5Y cells, reflecting the well‐established neurotoxic effect of this compound. However, treatment with the extract led to a considerable improvement in cell viability, suggesting a protective effect against MPP^+^‐induced cytotoxicity. These findings supported the hypothesis that HCE may counteract early cellular damage, potentially through mechanisms involving antioxidant and anti‐inflammatory pathways, which are further explored in subsequent analyses.

TH is a key enzyme involved in the synthesis of dopamine and is widely used as a marker of dopaminergic neuron integrity [31]. Its downregulation is commonly observed in PD and reflects the loss of dopaminergic function [31]. In contrast, α‐syn is a presynaptic protein whose abnormal accumulation and aggregation play a central role in PD pathology, contributing to the formation of Lewy bodies and promoting neurotoxicity [32]. Alterations in the expression of TH and α‐syn are therefore critical indicators of both neuronal damage and pathological protein misfolding [2, 33], making them essential markers for assessing the potential neuroprotective effects of therapeutic compounds.

In line with the established neurotoxic profile of MPP^+^, treatment of SH‐SY5Y cells resulted in a marked reduction in TH^+^ cells, indicating impaired dopaminergic function. This was accompanied by an increase in α‐syn^+^ cells, consistent with pathological protein accumulation observed in PD. Interestingly, co‐treatment with HCE significantly attenuated these effects, restoring TH levels and reducing α‐syn accumulation. These findings suggest a potential dual protective action of the extract: preserving dopaminergic phenotype and limiting pathological α‐syn aggregation. Such results strengthen the hypothesis that HCE may interfere with key molecular events in PD pathogenesis.

The NF‐κB signalling pathway plays a pivotal role in the regulation of neuroinflammation and has been related to PD progression [34]. In its inactive state, NF‐κB is sequestered in the cytoplasm by its inhibitor, IκBα [35]. Upon activation by inflammatory stimuli or cellular stressors, IκBα is phosphorylated and degraded, allowing NF‐κB to translocate to the nucleus and promote the transcription of pro‐inflammatory genes, including cytokines and chemokines [35]. This cascade contributes to a sustained inflammatory environment that exacerbates neuronal damage. Dysregulation of the NF‐κB/IκBα axis has been observed in various PD models and is considered a key driver of glial activation and dopaminergic neuron degeneration [36]. Therefore, targeting this pathway offers a promising strategy to mitigate inflammation‐associated neurotoxicity in PD.

Here, treatment with HCE exerted a notable modulatory effect on key components of the neuroinflammatory response. The extract preserved IκBα levels and reduced NF‐κB activation, indicating an inhibition of the inflammatory signalling pathway. In parallel, a significant downregulation of pro‐inflammatory cytokines like IL‐1β, IL‐17, and TNF‐α was observed. These cytokines are well‐known mediators of neuronal injury and glial activation in PD models, and their suppression suggests that the extract effectively reduces the inflammatory environment [2].

PPARγ is a nuclear receptor that regulates genes involved in inflammation, oxidative stress, and cell survival [37]. In recent years, it has gained increasing attention as a therapeutic target in neurodegenerative diseases, including PD [11, 38]. Activation of PPARγ, in fact, has been shown to attenuate neuroinflammatory responses [10], modulate glial cell activity, and protect dopaminergic neurons from degeneration [39, 40]. Of interest, several natural compounds exert their beneficial effects, at least in part, through PPARγ activation [41].

Based on this evidence, we hypothesised that the neuroprotective and anti‐inflammatory effects observed with HCE may be mediated, at least in part, by PPARγ activation. This prompted us to further investigate the involvement of this pathway in our experimental model. Consistent with our hypothesis, HCE treatment led to a significant upregulation of PPARγ expression in SH‐SY5Y cells. This suggests that the extract likely exerts its neuroprotective and anti‐inflammatory effects through activation of this nuclear receptor. The observed modulation of PPARγ is in line with the concurrent reduction in NF‐κB activity and pro‐inflammatory cytokine levels, supporting the idea of a mechanistic link between PPARγ activation and attenuation of neuroinflammation.

Oxidative and nitrosative stress are also key contributors to the pathophysiology of PD [42]. Impaired antioxidant defences and excessive production of ROS and RNS converge to create a toxic environment that damages lipids, proteins, and DNA [43]. In particular, elevated levels of NO and peroxynitrite have been implicated in promoting neuronal injury and amplifying neuroinflammatory responses [44]. This redox imbalance not only disrupts cellular homeostasis but also interacts with other pathological processes such as PD hallmarks and neuroinflammation [45].

The data obtained in this study indicate that HCE exerts a protective effect against both oxidative and nitrosative stress induced by MPP^+^ in SH‐SY5Y cells. The restoration of GSH levels and the upregulation of Cu/ZnSOD suggest an enhancement of the endogenous antioxidant defence system, which is typically compromised in Parkinsonian models. Furthermore, the observed reduction in ROS and ROMO‐1 levels supports the notion that the extract may also attenuate ROS generation.

In parallel, the decrease in 3‐NT and ${\mathrm{NO}}_{2}^{-}$ levels indicates a suppression of nitrosative stress, likely through inhibition of nitric oxide–related reactive species. Given the established role of oxidative and nitrosative stress in PD pathogenesis, these findings provide additional support for the neuroprotective profile of HCE. They also align with its observed anti‐inflammatory and pro‐survival effects, further suggesting that the extract's efficacy may derive from its multifaceted ability to modulate key pathological pathways.

Apoptotic cell death is a well‐documented feature of dopaminergic neuron loss in PD [46]. Various stressors, including the above mentioned mitochondrial dysfunction, oxidative damage, and inflammation, converge to activate intrinsic apoptotic pathways, ultimately leading to DNA fragmentation and neuronal demise [47]. The results from this study further support the neuroprotective effect of HCE, showing a clear reduction in MPP^+^‐induced apoptotic cell death. This finding suggests that the extract is able to counteract key pro‐apoptotic mechanisms triggered by mitochondrial dysfunction and oxidative stress.

Finally, to strengthen our hypothesis regarding the involvement of PPARγ in mediating all the effects of HCE observed in this study, we performed targeted gene silencing of PPARγ in SH‐SY5Y cells prior to treatment. This approach allowed us to directly assess whether the extract's anti‐inflammatory and antioxidant effects were dependent on this nuclear receptor. Following PPARγ silencing, the extract's ability to reduce key inflammatory markers including IL‐1β and IL‐17 as well as oxidative/nitrosative stress parameters such as 3‐NT and ${\mathrm{NO}}_{2}^{-}$ was markedly attenuated or abolished. These results provide compelling functional evidence that PPARγ is not merely associated with, but actively mediates, the protective actions of HCE. Thus, this silencing strategy significantly reinforces the proposed mechanism of action and validates PPARγ as a central player in the observed neuroprotection.

Our findings align with and extend recent evidence supporting the neuroprotective role of natural PPARγ agonists in models of PD. For instance, Wang and colleagues [48] proved that rutaecarpine, an alkaloid derived from traditional medicinal plants, exerts therapeutic effects in PD through PPARγ and lipid metabolism modulation. Similarly, Yang et al. [49] reported that abscisic acid, a plant‐derived phytohormone with structural similarities to thiazolidinediones, attenuates MPTP‐induced neuroinflammation via PPARγ and PGC‐1α upregulation.

These findings reinforce the central role of PPARγ as a molecular target for neuroprotection in PD and are consistent with our own results, in which the silencing of PPARγ significantly blunted the protective effects of HCE. Together, these studies support the therapeutic potential of natural compounds that activate PPARγ and highlight a promising pharmacological avenue that warrants further translational investigation.

## 5 Conclusion

Taken together, our findings demonstrate that HCE exerts neuroprotective effects in an in vitro model of PD through a multi‐targeted mechanism involving anti‐inflammatory, antioxidant, and anti‐apoptotic actions. A central role for PPARγ was functionally validated, as its silencing markedly reduced the extract's efficacy, highlighting this nuclear receptor as a key mediator. The modulation of critical markers such as NF‐κB, cytokines, oxidative/nitrosative stress indicators, and apoptotic signals suggests that HCE acts by restoring cellular homeostasis under neurotoxic conditions.

However, some limitations must be acknowledged. The study was conducted in a single in vitro model, which, while widely used, does not fully replicate the complex cellular interactions of the in vivo environment. Moreover, while the functional involvement of PPARγ was confirmed through knockdown experiments, further mechanistic studies are needed to elucidate the downstream signalling pathways in greater detail. Despite these limitations, our results strongly support the therapeutic potential of HCE in the context of neurodegeneration and reinforce the rationale for targeting PPARγ in PD.

## Author Contributions

Conceptualization: Marika Lanza. Methodology: Sarah Adriana Scuderi, Alessio Ardizzone, Deborah Mannino and Giovanna Casili. Validation: Alessio Ardizzone. Data curation: Sarah Adriana Scuderi, Alessio Ardizzone and Antonio Catalfamo. Writing – original draft preparation: Sarah Adriana Scuderi and Alessio Ardizzone. Writing – review and editing: Marika Lanza. Supervision: Marika Lanza and Emanuela Esposito. All authors have read and agreed to the published version of the manuscript.

## Funding

The authors have nothing to report.

## Ethics Statement

The authors have nothing to report.

## Consent

The authors have nothing to report.

## Conflicts of Interest

The authors declare no conflicts of interest.

## Supporting information


**Table S1:** jcmm71006‐sup‐0001‐TableS1.docx.

## Data Availability

The data supporting the findings of this study are available from the corresponding author upon reasonable request.
