# Supplementary material for: Aesculus hippocastanum Extract Exerts Neuroprotective Effects in an MPP+‐Induced Parkinson's Disease Model via PPARγ Activation
Source: J Cell Mol Med. 2026 Jan 5;30(1):e71006. doi: 10.1111/jcmm.71006 (PMC12771594; doi:10.1111/jcmm.71006)
Supplement: Supplementary file 1 — Table S1: jcmm71006‐sup‐0001‐TableS1.docx. [file JCMM-30-e71006-s001.docx]

| **Component** | **% *w/w*** |
| --- | --- |
| Escin | 19.55 % |
| Total flavonoids fraction | 2.84 % |
| Tannins | 3.06 % |
| Total Coumarins fraction | 0.91% |
| Polysaccharides and soluble sugars | 14.53 % |
| Inert plant material | 54.25 % |
| Residual moisture | 4.86 % |

**Supplementary Table 1.** Quali-quantitative composition of *Aesculus hippocastanum* dry extract per 100 g, and expressed as % *w/w*.
